# Combining inflammatory miRNA molecules as diagnostic biomarkers for depression: a clinical study

**DOI:** 10.3389/fpsyt.2023.1227618

**Published:** 2023-07-27

**Authors:** João Paulo Brás, Sara Pinto, Orlando von Doellinger, Joana Prata, Rui Coelho, Mário Adolfo Barbosa, Maria Inês Almeida, Susana Gomes Santos

**Affiliations:** ^1^i3S-Instituto de Investigação e Inovação em Saúde, Universidade do Porto, Porto, Portugal; ^2^INEB-Instituto de Engenharia Biomédica, Universidade do Porto, Porto, Portugal; ^3^ICBAS-Instituto de Ciências Biomédicas Abel Salazar, Universidade do Porto, Porto, Portugal; ^4^FMUP-Faculdade de Medicina, Universidade do Porto, Porto, Portugal; ^5^Departmento de Psiquiatria e Saúde Mental, Centro Hospitalar do Tâmega e Sousa, Penafiel, Portugal; ^6^Departamento de Psiquiatria e Saúde Mental, Centro Hospitalar Vila Nova de Gaia/Espinho, Vila Nova de Gaia, Portugal; ^7^Departmento de Neurociências Clínicas e Saúde Mental, Centro Hospitalar São João, Porto, Portugal

**Keywords:** depression, inflammation, MicroRNAs, PBMCs, cytokines

## Abstract

**Background:**

Inflammation has been implicated in core features of depression pathophysiology and treatment resistance. Therefore, new challenges in the discovery of inflammatory mediators implicated in depression have emerged. MicroRNAs (miRNAs) have been found aberrantly expressed in several pathologies, increasing their potential as biomarkers and therapeutical targets. In this study, the aim was to assess the changes and biomarker potential of inflammation-related miRNAs in depression patients.

**Methods:**

Depression diagnosis was performed according to the Diagnostic and Statistical Manual of Mental Disorders, Fifth Edition (DSM-5). 40 healthy controls and 32 depression patients were included in the study. The levels of inflammatory cytokines were measured in plasma, and expression levels of cytokines and inflammation-related miRNAs were evaluated in peripheral blood mononuclear cells (PBMCs).

**Results:**

Depression patients were found to have a pro-inflammatory profile in plasma, with significantly higher levels of TNF-α and CCL2 compared with controls. In PBMCs of depression patients, TNF-α and IL-6 expression levels were significantly up and downregulated, respectively. Moreover, miR-342 levels were found upregulated, while miR-146a and miR-155 were significantly downregulated. miR-342 expression levels were positively correlated with TNF-α. Importantly, when analyzed as a diagnostic panel, receiver operating characteristics (ROC) analysis of miR-342, miR-146a, miR-155 in combination, showed to be highly specific and sensitive in distinguishing between depression patients and healthy controls.

**Conclusion:**

In summary, these findings suggest that inflammation-related miRNAs are aberrantly expressed in depression patients. Moreover, we show evidences on the potential of the combination of dysregulated miRNAs as a powerful diagnostic tool for depression.

## 1. Introduction

Depression ranks as the most prevalent psychiatric disorder, with estimated over 280 million sufferers worldwide (WHO2023), and a lifetime prevalence of 10–20% (1). It also figures among the top three causes of disability worldwide and can affect individuals of all ages throughout their entire lifespan, with a higher prevalence in women (2). Conventional pharmacological treatments for depression are mainly based on the monoamines theory of depression, targeting neurotransmission regulation (3). However, it is estimated that between 30 to 50% of patients with major depression do not respond to the prescribed schemes of antidepressant medication (4–6), reinforcing the need to stratify patients, and understand the multifactorial etiology of this disorder. It is now clear that the major reason, still preventing a most accurate diagnosis as well as the development of better pharmacotherapies, is the poor understanding of the molecular pathology underlying depression. This leads to narrative and observation-based diagnosis, disregarding the biological particularities of each patient (7). Thus, there is an urgent need to establish complementary diagnostics tests, by defining diagnosis and prognosis biomarkers, as well as to develop a wider spectrum of novel therapeutics to target other possible underlying disease mechanisms.

Over the last decades, research has strongly focused on the inflammatory/immune hypothesis of depression, with most treatment resistant patients presenting a hyper-activation of the immune system (8–10). Clinical presentation of depression has long been compared with the so called “sickness behavior,” which occurs when individuals suffer from an inflammatory/infectious disease (11, 12). Moreover, the relation between depression and inflammation has been strongly suggested by patients with chronic inflammatory conditions, such as rheumatoid arthritis, psoriasis, inflammatory bowel disease and multiple sclerosis, among others (13–17). While the incidence of depression in patients with chronic inflammatory diseases is substantially higher, acute exacerbations of these diseases may also be preceded by stressful events or depressive episodes (9). Based on these findings, clinical trials have been exploring the potential of anti-inflammatory therapies to treat depression. While most of the reports suggest that anti-inflammatory agents play an efficient antidepressant role and are reasonably safe (18), others cast doubts on the potential therapeutic benefits of adjunctive anti-inflammatory drugs for the acute management of depression (19). Nonetheless, it is evident that a move away from symptom-based to a biological-based diagnosis is urgently needed. Innovative trial designs, with biologically based clinical outcomes, and more selective drugs, will prevent researchers from failing to take advantage of the increasing knowledge regarding the role of inflammation in depression.

In this sense, microRNAs (miRNAs) have recently emerged as important mediators in the pathophysiology of inflammation-related depression, with potential to be used as therapeutical targets and/or biomarkers (20–22). miRNAs are small non-coding RNAs that regulate multiple target transcripts, influencing entire gene networks in processes such as inflammation (inflammiRs), neurogenesis and neuronal plasticity (23–25). In this context, miRs like miR-145 and miR-146a have been recurrently associated with anti-inflammatory, neurogenesis and neuroprotective mechanisms (26–29), while miR-155 is known to have a dual role depending on the inflammatory stage, by acutely function as a strong promotor of anti-pathogen responses and lately limit the strength of the resulting NF-kB dependent inflammatory response (30, 31). Recently, our group found that miR-342 is upregulated *in vitro*, in tumor necrosis factor-α (TNF-α) activated microglia (32), and *in vivo*, in the hippocampus of rats exhibiting depressive-like behaviors, increased hippocampal TNF-α expression and microglia activation (33). Based on these features, we hypothesize that these inflammiRs may appear dysregulated in depression, with strong genetic support for associating them and their targets with this condition.

In this study, we evaluated the levels of inflammatory cytokines in plasma and peripheral blood mononuclear cells (PBMCs), and the expression levels of miR-145, miR-146a, miR-155 and miR-342 in PBMCs. The results show a pro-inflammatory profile in plasma of depression patients, with increased TNF-α and CCL2, as well as significant correlations between the levels of different pro-and anti-inflammatory mediators. In PBMCs, TNF-α and IL-6 expression levels were significantly up and downregulated, respectively. Also, miRNA-342 was found significantly upregulated, while miRNA-155 and miRNA-146a were significantly downregulated in depression patients compared with healthy controls. The levels of these miRNAs corelated with those of the inflammatory cytokines, and the receiver operating characteristics (ROC) analysis of their levels, showed higher significance and area under the curve, when miRNA-342, miRNA-155 and miRNA-146a were considered together. In summary, the results presented here, open new possibilities for the use of miRNA panels as depression biological biomarkers.

## 2. Materials and methods

### 2.1. Ethics statement

All obtained human samples and procedures were performed in agreement with the principles of the Declaration of Helsinki. Blood samples were collected from patients enrolled at the psychiatry departments of *Centro Hospitalar do Tâmega e Sousa, EPE* and *Centro Hospitalar de Vila Nova de Gaia/Espinho, EPE*; and from healthy blood donors, at *Serviço de Imunohemoterapia*, *Centro Hospitalar Universitário de São João*, after informed consent. All experimental protocols were conducted following the approval and recommendations of the Ethics Committees for the hospitals involved.

### 2.2. Experimental design

Patients admitted to the Department of Psychiatry and Mental Health of *Centro Hospitalar do Tâmega e Sousa* (Penafiel, Portugal) and outpatients from the Department of Psychiatry and Mental Health of *Centro Hospitalar Vila Nova de Gaia/Espinho* (Vila Nova de Gaia, Portugal), admitted to the hospitals with a major depression episode and that agreed to take part, were enrolled in the study. Participants ranged in age from 18–65 years and entered the study after screening and diagnosis of major depressive disorder was confirmed by two psychiatrists, as described previously (6, 10). Briefly, screening included a structured clinical interview to assess the presence of major psychiatric syndromes according to the Diagnostic and Statistical Manual of Mental Disorders, Fifth Edition (DSM-5), an assessment of current psychiatric symptoms, and a determination of previous antidepressant treatment. Blood samples were obtained from depression patients at the time they were admitted to hospital. Single blood samples were also obtained from healthy control subjects at the *Serviço de Imunohemoterapia* do *Centro Hospitalar Universitário de São João* (Porto, Portugal). Healthy control subjects were screened to rule out a personal or family history (first-degree relative) of psychiatric disorder. Patients and controls were excluded from the study if presenting any of the following: (i) psychotic symptoms; (ii) presence of an infectious or inflammatory illness or the regular use of anti-inflammatory medication; (iii) Inability to completely understand and fill in the self-assessment instruments; (iv) Being part of another study or with other psychological/psychopharmacological treatment.

### 2.3. Plasma and PBMCs isolation

Peripheral blood was collected using VACUETTE^®^ Tubes EDTA K3 (Greiner Bio-One, France). Blood components were separated by centrifuging at 1200 g, for 20 min, at room temperature (RT), without break. Plasma was collected and centrifuged twice at 2500 g, for 10 min at 4°C before being aliquoted and stored at-80°C. Medial layer containing PBMCs was slowly collected and transferred into a new 15 mL centrifuge tube. PBMCs were diluted in an equal volume of PBS 1x, slowly layered over Lymphoprep™ (Ratio 1:1, StemCell Technologies, Canada) and centrifuged at 800 g, for 20 min, at RT, without break. Medial layer containing enriched PBMCs was collected and cells washed twice with PBS 1x (300 g, 10 min, at 4°C) before being lysed with TRIzol^®^ (Invitrogen, MA, United States).

### 2.4. RNA extraction

Total RNA was extracted using TRIzol^®^ according to the manufacturer’s instructions. RNA concentration and purity were evaluated in a NanoDrop 1,000 (ThermoFisher Scientific, MA, United States). Ratios of 260/280 and 260/230 nm ranged between 1.8 and 2.2. RNA integrity was evaluated by agarose gel electrophoresis or by Experion™ automated electrophoresis system (Bio-Rad, CA, United States).

### 2.5. Reverse transcription and real-time quantitative polymerase chain reaction (RT-qPCR)

For gene expression analysis, RNA was treated with TURBO DNA-free Kit (Invitrogen) and cDNA was synthesized using Random Hexamers (Invitrogen), dNTPs (Bioline, OH, United States) and SuperScript^®^ III Reverse Transcriptase (Invitrogen). qPCR was carried out in CFX96 Touch™ Real-Time PCR Detection System (Bio-Rad) using cDNA, primers and iQ SYBR Green Supermix (Bio-Rad). GAPDH was used as reference gene. Oligonucleotides used for qPCR experiments are shown in Supplementary Table S1.

miR-145-5p, miR-146a-5p, miR-155-5p and miR-342-3p expression was evaluated using TaqMan miRNA assays (Applied Biosystems, MA, United States). Briefly, cDNA was synthesized using 30 ng of RNA as a template, gene-specific stem-loop Reverse Transcription primer, and the TaqMan microRNA reverse transcription kit (Applied Biosystems). qPCR was carried out in CFX384 Touch™ Real-Time PCR Detection System (Bio-Rad) using cDNA, TaqMan probe and SsoAdvancedTM Universal Probes Supermix (Bio-Rad). Small nuclear RNA U6 was used as reference gene. All runs were performed in duplicate. Relative expression levels were calculated using the quantification cycle (Cq) method, according to MIQE guidelines (34).

### 2.6. Enzyme-linked immunosorbent assay (ELISA)

CCL2, IL-6, IL-1β, TNF-α, IL-4 and IL-10 levels were evaluated by ELISA, according to the manufacturer’s instructions (ELISA MAX™ Deluxe Set, BioLegend, CA, United States). Absorbance was measured in a plate reader at 450 nm, with wavelength correction at 570 nm. Cytokine concentrations (pg/mL) were determined using a standard calibration curve.

### 2.7. Statistical analysis

Statistical analysis was performed using GraphPad Prism version 7 (GraphPad Software, Inc.). Gaussian distribution was tested by the D’Agostino & Pearson and the Shapiro–Wilk normality tests. For non-normal distribution data, statistical differences were evaluated by unpaired Mann–Whitney rank test. When data passed normality test, unpaired T-tests were performed. Spearman correlation analyses (non-parametric data) between plasma cytokines, cytokine mRNA levels and miRNA levels in PBMCs were performed considering only depression subjects. The total number of individuals and statistical tests used are identified in each figure legend. Statistical significance was considered for *p* < 0.05 (^*^ *p* < 0.05, ^**^ *p* < 0.01, ^***^ *p* < 0.001, n.s.: non-significant). The diagnostic value of the tested miRNAs (individually or combined) was calculated using receiver operating characteristics (ROC) curve in GraphPad. For that, miRNAs relative expression values were previously combined using the binary logistic toll on SPSS considering the experimental group as the dependent variable and the different tested miRNAs as covariates. The area under the curve (AUC) measured the ability of each miRNA or the miRNA panel to distinguish between both groups, and the value of p tested the null hypothesis that the AUC equals 0.5.

## 3. Results

### 3.1. Depression patients show increased levels of inflammatory mediators in plasma

A total of 72 subjects were included in the study. The depression group was composed of 32 subjects (27 females and 5 males) with a mean age of 38.81 ± 2.13, while 40 healthy subjects (23 females and 17 males), with a mean age of 37.95 ± 1.71, were included in the control group (Supplementary Table S2).

**Figure 1:**
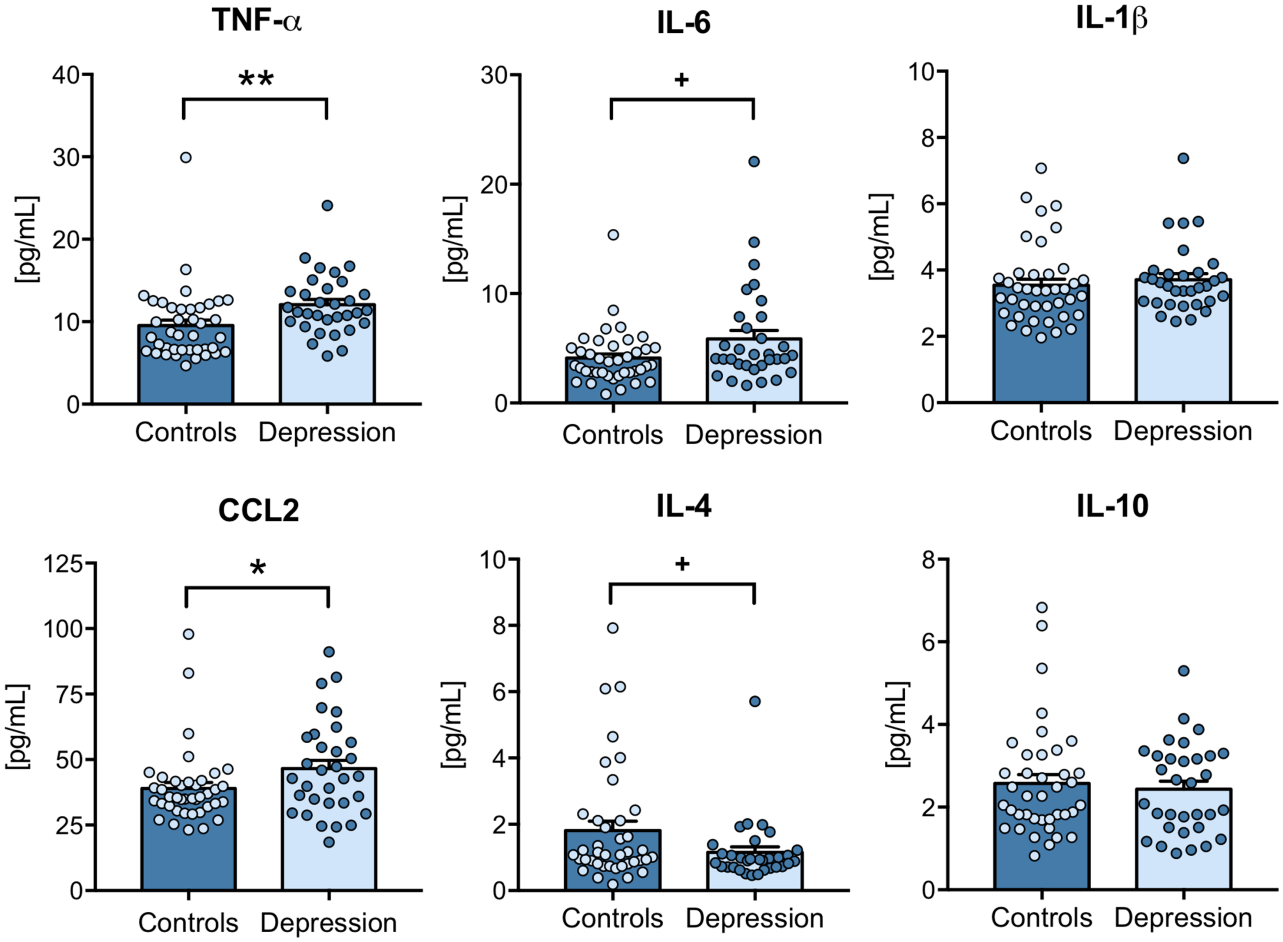
Plasma levels of inflammatory cytokines in depression patients versus healthy controls. Blood components were separated, and plasma was used to measure CCL2, IL-6, IL-1β, TNF-α, IL-4 and IL-10 levels by ELISA, according to the manufacturer’s instructions. All samples were tested simultaneously and under the same conditions for each cytokine. Cytokine concentrations [(pg/mL)] were determined using a standard calibration curve, and results are presented as mean ± SEM. Each dot represents an individual (control or depression patient). Statistical differences between groups were evaluated using Mann–Whitney non-parametric unpaired test (^***^ *p* < 0.001, + *p* < 0.1). N: Healthy controls = 40; Depression patients = 32.

Dysregulated levels of inflammatory mediators have been described in depression patients (8), so the plasma levels of several inflammatory markers were evaluated by ELISA. Results revealed that the classical pro-inflammatory cytokine TNF-α (*p* = 0.0011) and also the CCL2 chemokine (*p* = 0.0453), were significantly upregulated in depression patients compared with healthy controls (Figure 1). Although not significantly altered, IL-6 levels tend to be upregulated (*p* = 0.0691) and IL-4 downregulated (*p* = 0.0746) in depression patients (Figure 1). Plasma levels of IL-1β and IL-10 were not significantly different between depression patients and healthy controls (Figure 1). Next, Spearman correlations were used to understand the correlation between the levels of these different inflammatory mediators. The results revealed that depression patients’ plasma levels of CCL2, IL-6, IL-4 and IL-10 were all positively correlated (Supplementary Table S3).

### 3.2. TNF-α expression is increased in PBMCs of depression patients

**Figure 2:**
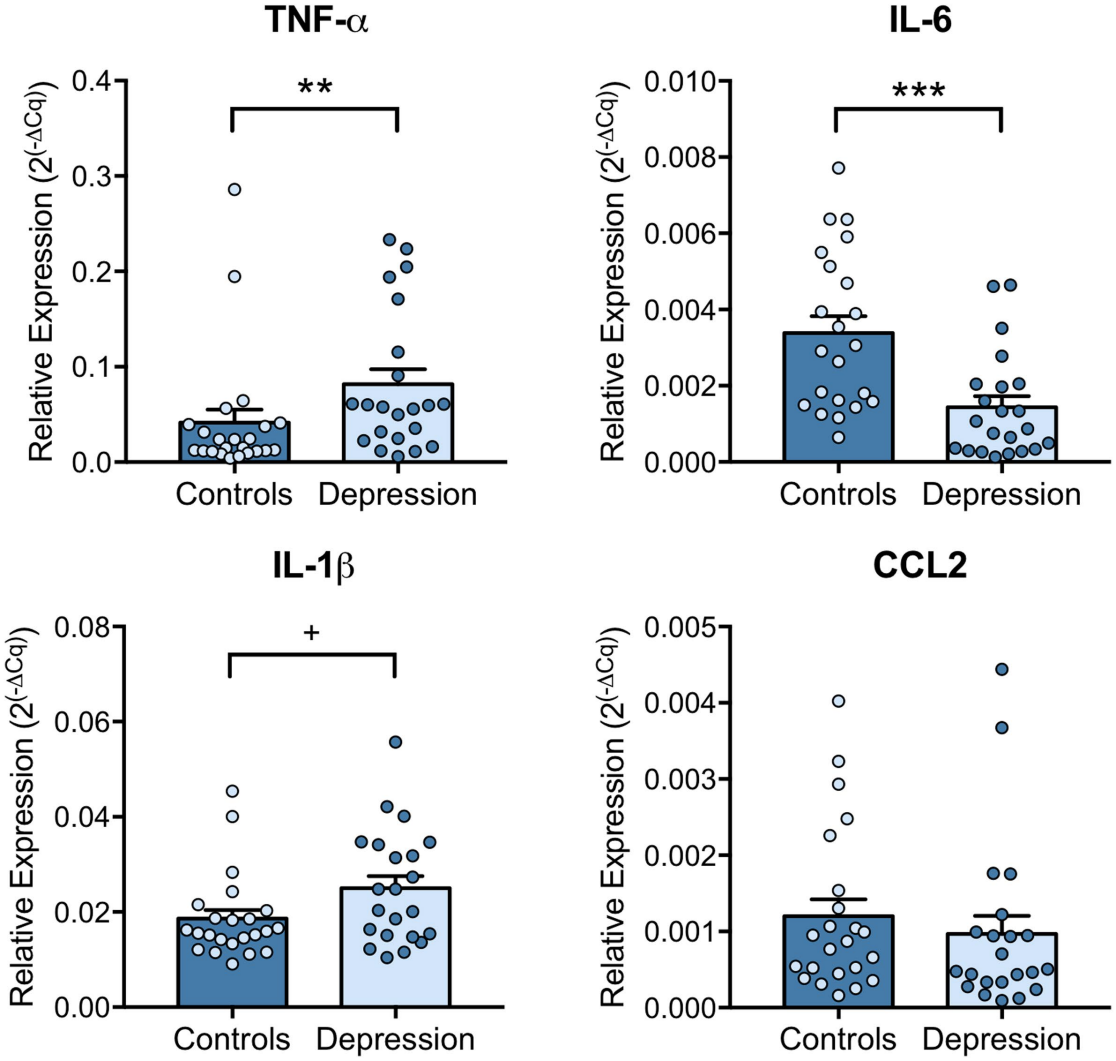
Levels of pro-inflammatory cytokines in PBMCs of depression patients versus healthy controls. Blood components were separated, PBMCs collected, and RNA extracted using TRIzol. CCL2, IL-6, IL-1β, TNF-α mRNA levels in PBMCs were evaluated by RT-qPCR using GAPDH as internal control. Relative expression levels were calculated using the quantification cycle (Cq) method, according to MIQE guidelines, and results are presented as mean ± SEM. Each dot represents an individual (Control or Depression patient). Statistical differences between groups were evaluated using Mann–Whitney non-parametric unpaired test (^***^ *p* < 0.001, ** *p* < 0.01 and + *p* < 0.1). N: Healthy controls = 23; Depression patients = 22.

To further explore the contribution of circulating immune cells to the changes observed on inflammatory markers, their mRNA levels were evaluated in PBMCs by RT-qPCR. Results revealed that, in agreement with the increased plasma levels, TNF-α mRNA levels were significantly upregulated (*p* = 0.0073). Interestingly, while protein levels of IL-6 in plasma showed a tendency for increase, its mRNA levels in PBMCs were significantly downregulated (*p* < 0.001) in depression patients compared with healthy controls (Figure 2). Although IL-1β mRNA levels tend be upregulated in depression patients (*p* = 0.0606, Figure 2), no significant differences were found between the mRNA levels of IL-1β nor CCL2 in PBMCs of depression patients and healthy controls. Interestingly, a positive Spearman correlation was maintained at the mRNA level in PBMCs of depression patients, between CCL2, IL-6 and TNF-α (Supplementary Table S4).

### 3.3. Correlation between miRNAs and inflammatory cytokines in PBMCs of depression patients

**Figure 3:**
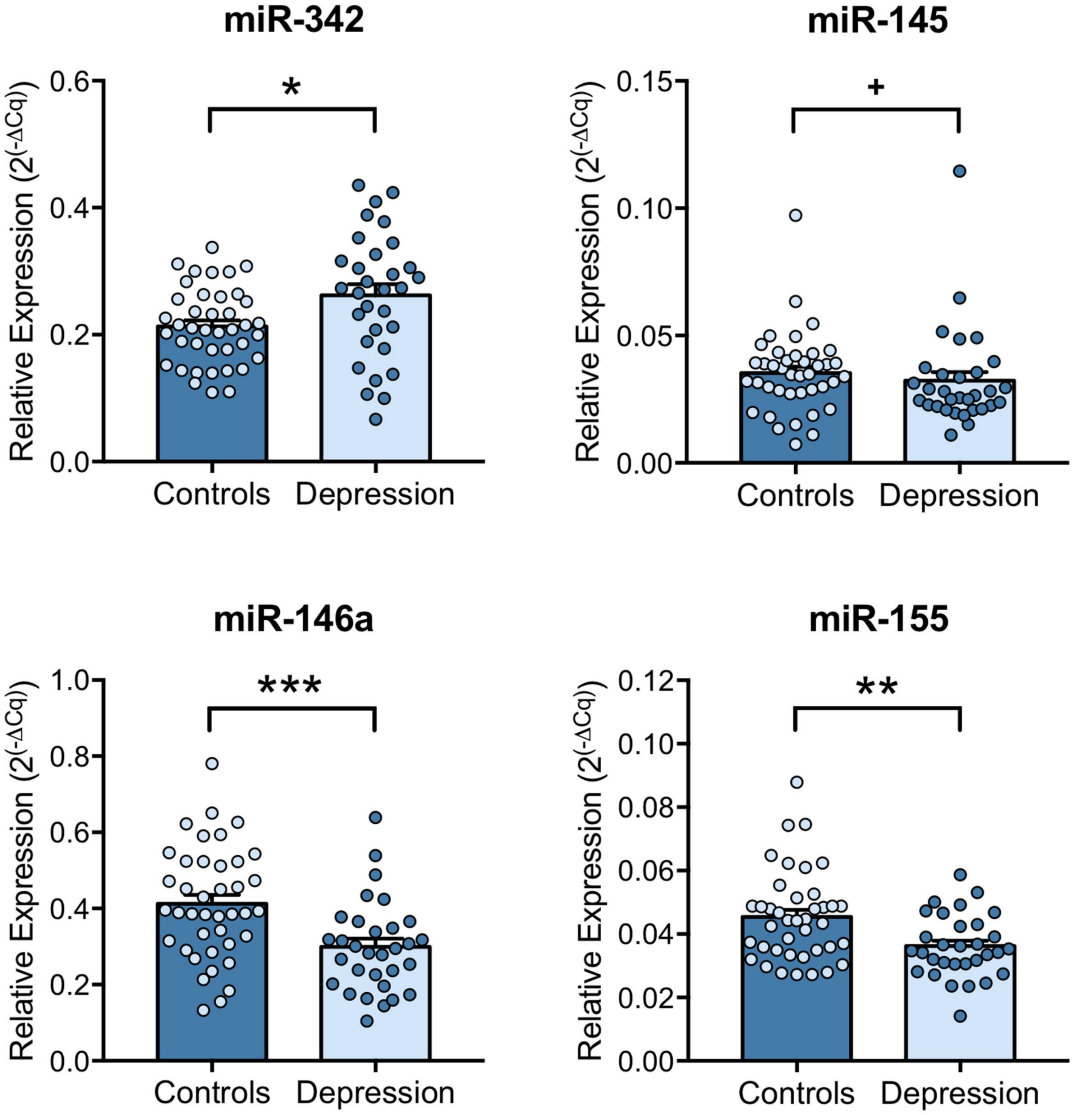
miRNA expression levels in PBMCs of depression patients versus healthy controls. miR-342, miR-145, miR-146a and miR-155 expression levels in PBMCs were evaluated by RT-qPCR using U6 snRNA as internal control. Relative expression levels were calculated using the quantification cycle (Cq) method, according to MIQE guidelines, and results are presented as mean ± SEM. Each dot represents an individual (Control or Depression patient). Statistical differences between groups were evaluated using Mann–Whitney non-parametric unpaired test or unpaired T-test (^**^ *p* < 0.01, * *p* < 0.05 and + *p* < 0.1). N: Healthy controls = 40; Depression patients = 32.

Next, the regulation of inflammatory markers was explored, by investigating the levels of several miRNAs that can target inflammatory mediators, like TNF-α. miR-342, miR-145, miR-146a and miR-155 expression levels in PBMCs of depression patients and healthy controls were evaluated by RT-qPCR. Results revealed that miR-342 is significantly upregulated (*p* = 0.0117), while miR-146a (*p* < 0.001) and miR-155 (*p* = 0.0056) levels are significantly downregulated in PBMCs of depression patients compared with healthy controls (Figure 3). miR-145 expression levels tend to be downregulated in PBMCs of depression patients, but this decrease is not statistically significant (*p* = 0.067, Figure 3). Spearman correlation analysis showed that TNF-α mRNA levels were significantly positively correlated with miR-342 (*r* = 0.4274, *p* = 0.0472), while IL-6 mRNA levels were significantly correlated with miR-145 levels (*r* = 0.4850, *p* = 0.0221). On the other hand, IL-1β mRNA levels were negatively correlated with miR-155 levels (−0.5697, *p* = 0.0056, Table 1). No statistically significant correlations were observed between the expression levels of the tested miRNAs in PBMCs of depression patients (Supplementary Table S5).

**Table 1:** Spearman correlations between cytokines mRNA levels and miRNA levels in PBMCs of depression patients.

		PBMCs							
		miR-342		miR-145		miR-146a		miR-155	
		*r*	*p*-value	*r*	*p*-value	*r*	*p*-value	*r*	*p*-value
**PBMCs**	**CCL2**	0.3145	0.1539	0.4150	0.0547	0.2275	0.3084	0.2320	0.2986
	**IL-6**	0.3642	0.0956	**0.4850**	**0.0221**	−0.0254	0.9106	0.0592	0.7932
	**IL-1β**	−0.2795	0.2077	0.0367	0.8711	−0.1383	0.5392	**−0.5697**	**0.0056**
	**TNF-α**	**0.4274**	**0.0472**	0.3879	0.0744	−0.0909	0.6874	0.1507	0.5030

The coefficient *r* and *p*-value for each correlation are presented. Statistically significant correlations are bolded (*p* < 0.05).

**Figure 4:**
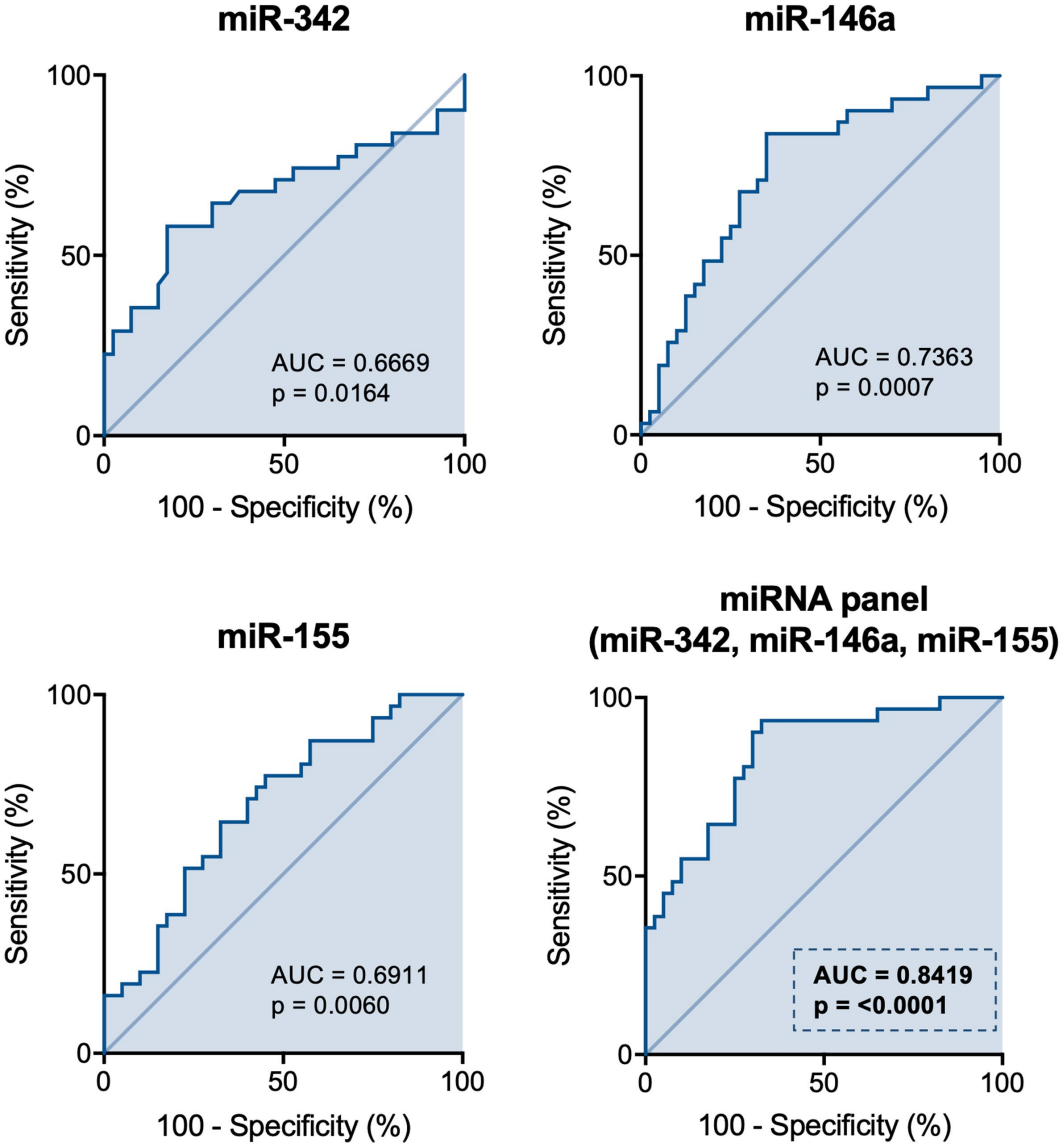
ROC analysis of the differently expressed miRNAs. ROC analysis was performed to evaluate the sensitivity of miR-342, miR-146a and miR-155 to distinguish between depression patients and controls when used individually or in combination as a miRNA panel. The area under the curve (AUC) represents the measure of the ability of each miRNA or the miRNA panel to distinguish between both groups, and the value of p tests the null hypothesis AUC equals 0.5. N: Healthy controls = 40; Depression patients = 32.

Finally, the potential for these miRNAs to be used as biological quantitative diagnostic markers for depression was explored. ROC analysis was performed to evaluate the ability of the differently expressed miRNAs to distinguish between depression patients and healthy controls, individually or in combination. The area under the curve (AUC) for miR-342, miR-146a and miR-155 when tested individually was 0.667 (CI = 0.53–0.80, *p* = 0.0164), 0.736 (CI = 0.62–0.85, *p* = 0.0007) and 0.691 (CI = 0.57–0.81, *p* = 0.006) respectively. When tested as a miRNA panel, sensitivity (80.6%) and specificity (72.5%) increased significantly and the use of the three miRNAs in combination was the best classifier (AUC = 0.842, CI = 0.75–0.93, *p* < 0.0001; Figure 4 and Supplementary Table S6).

## 4. Discussion

In this study, we evaluated whether the levels of inflammatory cytokines and the expression levels of miR-145, miR-146a, miR-155 and miR-342 in PBMCs, were altered in depression patients, compared with healthy controls. Depression patients were found to have significantly higher levels of TNF-α and CCL2 in the plasma, and increased expression levels of TNF-α together with decreased IL-6, in PBMCs. Moreover, miR-342 levels were found upregulated, while miR-146a and miR-155 were significantly downregulated in PBMCs of depression patients. Of note, expression levels of miR-342 positively correlated with those of TNF-α in PBMCs. Importantly, when these three miRNAs were analyzed as diagnostic panel, their ROC analysis, showed to be highly significant, with an area under the curve of 0.8419.

In recent years, the immune system has emerged as a key player in depression symptomatology and treatment resistance (35). A segment of depression patients exhibits chronic inflammation, shown by increased systemic levels of inflammation-related markers, including cytokines and other inflammatory mediators, such as C-Reactive Protein (CRP), which have been correlated with more severe depression symptoms (8, 36). In this study, we found an upregulation of TNF-α and CCL2 levels in the plasma of depression patients. These findings are in accordance with the literature, as TNF-α and CCL2 have been recurrently found upregulated in depression patients, either at baseline and in treatment-resistant patients (8, 37). On the other hand, plasma levels of IL-6 and IL-1β were not significantly upregulated in the segment of patients analyzed. Despite being frequently dysregulated in inflammation-related disorders, IL-1β has not been consistently found increased in depression patients (38). In the case of IL-6, literature has been supporting an increase in plasma of depression patients (39). Although there is a tendency to be upregulated, no significant differences were found for IL-6 in depression patients versus healthy controls, in the current study. Regarding anti-inflammatory cytokines IL-4 and IL-10 were unchanged between depression patients and controls. A recent report by Yuan et al., assessing reproducibility and specificity of inflammation-related markers in major psychiatric disorders, also found no changes in plasma levels of IL-4 and reported inconsistent findings on the levels of IL-10 in depression (38).

Inflammation is known to play a role in core features of depression, particularly due to the action of central nervous system (CNS) activated microglia and astrocytes, infiltrated and peripheral BMCs (particularly monocytes/macrophages and T lymphocytes), as well as the immunoreactive molecules (e.g., cytokines and chemokines) they release (40, 41). Since brain tissue is rarely available for study, the analysis of other peripheral information sources, such as saliva, plasma, serum and particularly PBMCs, has received increasing attention. In fact, previous studies have shown that peripheral blood cells share more than 80% of the transcriptome with brain tissue, therefore offering a potential diagnostic tool that can dynamically reflect changes in brain macro-and micro-environments (42). In this sense, we analyzed the expression levels of several inflammatory molecules in PBMCs of both depression and healthy subjects. In line with what was observed in plasma, TNF-α mRNA levels were found upregulated in depression patients compared with healthy controls, suggesting that PBMCs might be contributing to the observed increased levels in plasma. On the contrary, while CCL2 levels in plasma were found upregulated in depression patients, mRNA levels of this cytokine in PBMCs remained unaltered. Moreover, despite a tendency to an increase in plasma, IL-6 mRNA levels were found downregulated in the PBMCs of depression patients, in what we hypothesize could be a mechanism of negative feedback, to compensate the increased levels of IL-6 and other dysregulated cytokines in plasma. In fact, this disconnection between cytokine plasma levels and circulating inflammatory cells phenotype was also reported before (43). Hasselmann et al. found that despite depression patients showing higher frequency and higher absolute numbers of non-classical monocytes, there was no correlation between those changes and circulating levels of CRP, IL-6, IL-1β, or TNF-α (43). Although being frequently overlapped, these findings support the need to distinguish between cytokine levels measured in plasma/serum and mRNA levels detected in circulating inflammatory cells, in order to fully understand the contribution of immune cells and the cascade of activation states they go through during chronic systemic inflammation. In the current study, patients with reported inflammation and/or infection, and/or the use of anti-inflammatory medication were not included. The CRP levels were not measured and used as an exclusion criteria for any inflammation or infection, as this protein has been found significantly upregulated in a subgroup of depression patients (8).

miRNAs’ unique expression patterns and ability to modulate mRNA levels of a large number of target genes, often related to disease-associated pathological processes, such as in inflammation-related depression, increases their desirability as diagnostic markers (44–46). In this study, an overexpression of miR-342 and a downregulation of miR-146a and miR-155 in the PBMCs of depression patients were found. Previously, our group have elucidated the role of miR-342 as a crucial mediator of TNF-α-driven microglia activation (32) and identified it as being upregulated in the hippocampus of rats exhibiting depressive-like behaviors (33). *In vitro*, after being found upregulated in TNF-α stimulated microglia, miR-342 microglial overexpression *per se* was shown to be sufficient to induce neurotoxicity, activating the NF-kB pathway, and leading to increased microglial secretion of TNF-α and IL-1β, whereas *in vivo* increased hippocampal levels of miR-342 were positively correlated with TNF-α expression, microglia activation and depressive-like behaviors (33). In fact, microglia activation has been associated with neurodegenerative diseases and psychiatric disorders, including depression (47). In response to adverse stimuli, such as psychological stress, brain injuries or infections, microglia overproduce proinflammatory cytokines and chemokines that not only influence the surrounding microenvironment but also promote the recruitment of peripheral immune cells (48, 49). This results in exacerbated neuroinflammation, leading to an imbalance of several brain functions, some of which characteristic of depression (47, 50). To our knowledge, this is the first study reporting increased expression levels of miR-342 in depression patients. Importantly, miR-342 levels were positively correlated with TNF-α levels, showing that a strong interplay between TNF-α and miR-342 is also found in humans, and outside the brain in circulating cells. miR-146a has been shown to attenuate deleterious processes associated with dysregulated inflammation in several diseases, including rheumatoid arthritis and atopic dermatitis (51). miR-146a acts as a mitigator of inflammatory responses by targeting key molecules of NF-kB and JAK–STAT signaling pathways, thereby reducing pro-inflammatory cytokines production, such as TNF-α and IL-8 (52, 53). In pathologies with chronic low-grade baseline inflammation, miR-146a have been recurrently found downregulated (54–56), indicating that its dysregulation contributes to pathology. Previously, miR-146a was found downregulated in the prefrontal cortex of suicide victims diagnosed with clinical depression (57). Here, we show that miR-146a levels are also downregulated in the PBMCs of depression patients, which may partially explain the observed increase of TNF-α levels. In agreement, a recent study performed by Hung et al. evaluating the expression levels of intracellular miRNAs that regulate TLR4 signaling in PBMCs and monocytes of depression patients, found a downregulation of miR-146a and miR-155 in PBMCs (58). In the current study, we also found miR-155 levels downregulated in PBMCs of depression patients. miR-155 is known as a master regulator of inflammation performing both pro-and anti-inflammatory functions (59). miR-155 is normally found upregulated in acute inflammatory responses as its expression is highly induced by TLR ligands/activation (60). In early inflammatory responses stages, miR-155 targets the suppressor of cytokine signaling 1 (SOCS1), a key molecule of the classical negative feedback system that regulates cytokine signal transduction (61). In turn, when inflammation is chronically exacerbated, miR-155 overexpression attenuates inflammation intensity by targeting key TLR-signaling downstream molecules (62). Specifically, by targeting NF-kB p65, miR-155 overexpression has been shown to serve as a negative feedback regulator of inflammation, reducing TNF-α production (63, 64). Thus, we hypothesize that the upregulation of TNF-α levels in plasma and PBMCs of depression patients may result from a combined increase of miR-342, a TNF-α promoter, and downregulation of miR-155 and miR-146a, TNF-α negative regulators. Importantly, ROC analysis revealed that, when used in combination, the expression levels of miR-342, miR-146a and miR-155, constitute a diagnostic panel with increased sensibility and specificity. The combination of miRNAs in panels has been shown to increase their accuracy and diagnosis value, when compared to the use of single miRNAs (65).

In this study the systemic changes in the cytokine profile and inflammiRs were analyzed, but other important mediators, like those of the neuroendocrine system (e.g., cortisol), or other inflammatory mediators (e.g., CRP) were not evaluated, which is a limitation of the study and should be addressed in the future.

## 5. Conclusion

Globally, we show that depression patients have increased systemic inflammation, reflected on increased plasma levels of TNF-α and CCL2, increased TNF-α mRNA levels in PBMCs, and dysregulated expression of key and inflammation-related miRNAs in PBMCs. Future work should investigate the potential use of miR-342, miR-146a and miR-155 as a miRNA panel to diagnose depression and monitor treatment response, particularly in cases with exacerbated baseline inflammation.

## Data availability statement

The original contributions presented in the study are included in the article/Supplementary material, further inquiries can be directed to the corresponding author.

## Ethics statement

The studies involving human participants were reviewed and approved by Ethics committees of the “Centro Hospitalar do Tâmega e Sousa, EPE,” “Centro Hospitalar de Vila Nova de Gaia/Espinho, EPE”; and “Centro Hospitalar Universitário de São João.” The patients/participants provided their written informed consent to participate in this study.

## Author contributions

JB, SP, OD, JP, RC, MB, MA, and SS discussed patient selection criteria and revised the manuscript. JB processed samples, treated results and drafted the manuscript. SP collected samples and patient data, treated results and drafted the manuscript. OD, JP, and RC established inclusion or exclusion criteria, interviewed patients, collected and interpreted the data. MB, MA, and SS helped with sample processing, data analysis and interpretation and manuscript writing. All authors contributed to the article and approved the submitted version.

## Funding

This work was funded by project NORTE-01-0145-FEDER-000012, supported by Norte Portugal Regional Operational Programme (NORTE 2020), under the PORTUGAL 2020 Partnership Agreement, through the European Regional Development Fund (ERDF). This work was also supported by BIP proof, UP. The funder was not involved in the study design, collection, analysis, interpretation of data, the writing of this article, or the decision to submit it for publication.

## Conflict of interest

The authors declare that the research was conducted in the absence of any commercial or financial relationships that could be construed as a potential conflict of interest.

## Publisher’s note

All claims expressed in this article are solely those of the authors and do not necessarily represent those of their affiliated organizations, or those of the publisher, the editors and the reviewers. Any product that may be evaluated in this article, or claim that may be made by its manufacturer, is not guaranteed or endorsed by the publisher.

## Supplementary material

The Supplementary material for this article can be found online at: https://www.frontiersin.org/articles/10.3389/fpsyt.2023.1227618/full#supplementary-material
