# Supplementary material for: Combining inflammatory miRNA molecules as diagnostic biomarkers for depression: a clinical study
Source: Front Psychiatry. 2023 Jul 27;14:1227618. doi: 10.3389/fpsyt.2023.1227618 (PMC10413105; doi:10.3389/fpsyt.2023.1227618)

**Supplementary Information**

**Content:**

*Table S1*

*Table S2*

*Table S3*

*Table S4*

*Table S5*

*Table S6*

**Table S1. Oligonucleotides sequences for RT-qPCR.** Oligonucleotides used to amplify human mRNAs encoding inflammatory markers and reference gene, based on GenBank sequences. Abbreviations: IL1B, interleukin 1 beta; IL6, interleukin 6; TNF, tumor necrosis factor; CCL2, C-C motif chemokine ligand 2; GAPDH, glyceraldehyde 3-phosphate dehydrogenase.

| **Gene** | **Accession Number** | **Forward (5’-3’)** | **Reverse (5’-3’)** |
| --- | --- | --- | --- |
| IL1B | NM_000576.3 | CTTCAGCCAATCTTCATT | CACTGTAATAAGCCATCAT |
| IL6 | NM_000600.5 | AATTCGGTACATCCTCGACGG | GGTTGTTTTCTGCCAGTGCC |
| TNF | NM_000594.4 | TCTCTCTAATCAGCCCTCTG | TGCTACAACATGGGCTACAG |
| CCL2 | NM_002982.4 | CAGCCAGATGCAATCAATGC | GCACTGAGATCTTCCTATTGGTGAA |
| GAPDH | NM_002046.7 | CCATCCACAGTCTTCTGGGT | CCTCAAGATCATCAGCAAT |

**Table S2. Demographic characteristics of depression patients and healthy controls groups.**

|  | **Depression** | **Controls** |
| --- | --- | --- |
| **N** | 32 | 40 |
| **Sex (F/M)** | 27/5 | 23/17 |
| **Age ± SEM** | 38.81 ± 2.13 | 37.95 ± 1.71 |

**Table S3. Spearman correlations between plasma cytokines in depression patients.** The coefficient r and p-value for each correlation are presented. Statistically significant correlations are highlighted (p<0.05).


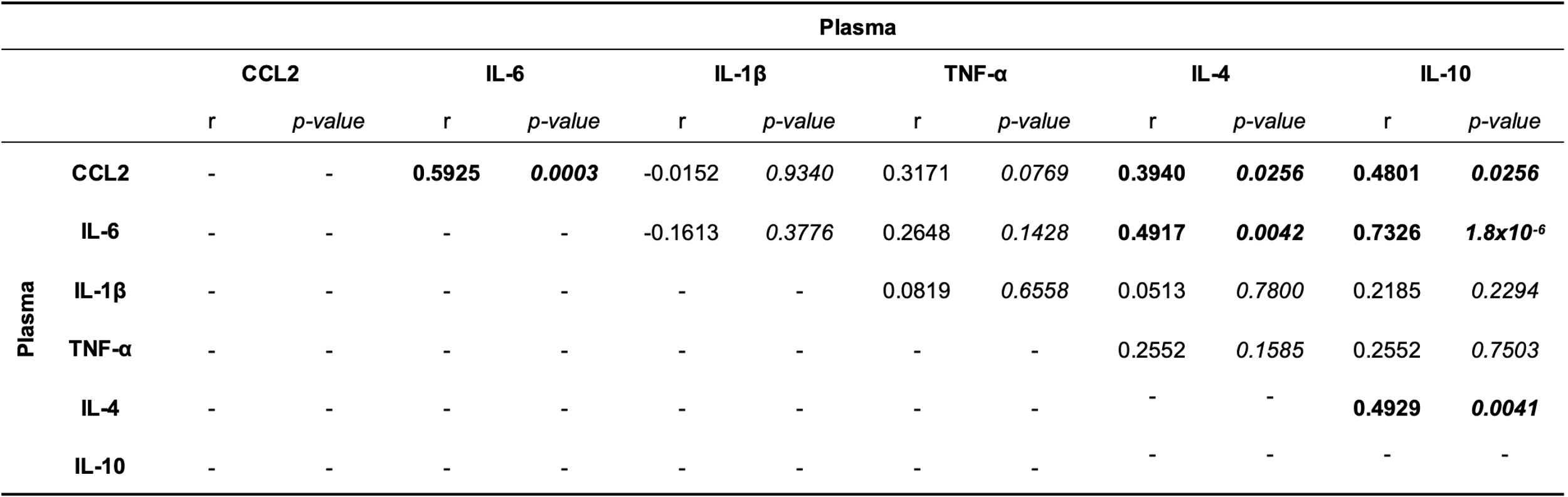


**Table S4. Spearman correlations between cytokines mRNA levels in PBMCs of depression patients.** The coefficient r and p-value for each correlation are presented. Statistically significant correlations are highlighted (p<0.05).


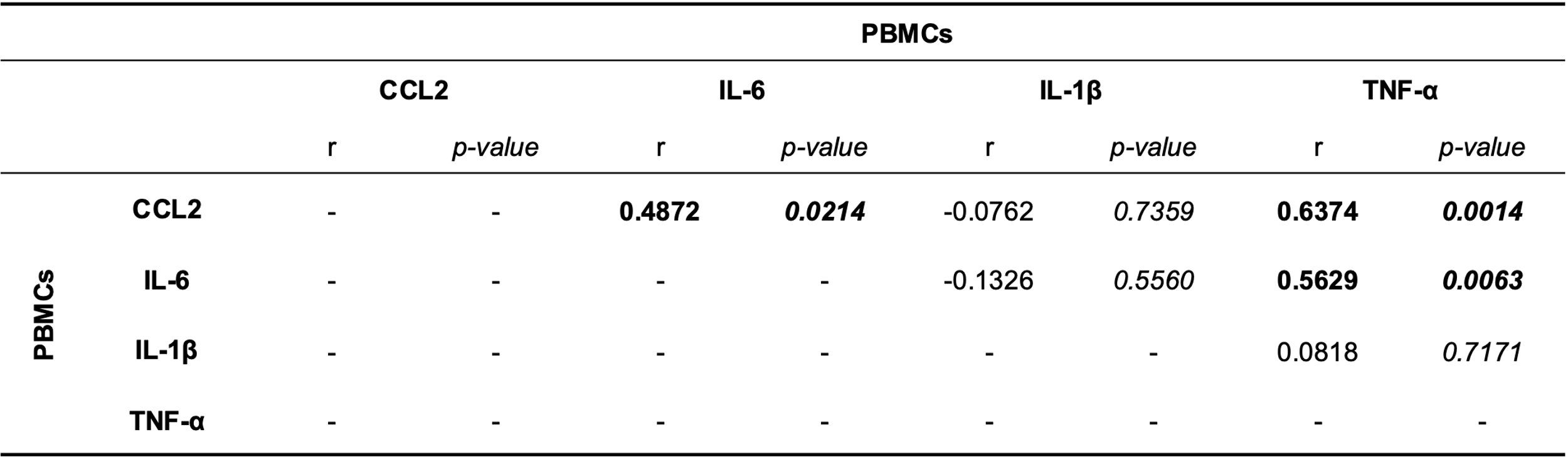


**Table S5. Spearman correlations between miRNA levels in PBMCs of depression patients.** The coefficient r and p-value for each correlation are presented. Statistically significant correlations are highlighted (p<0.05).


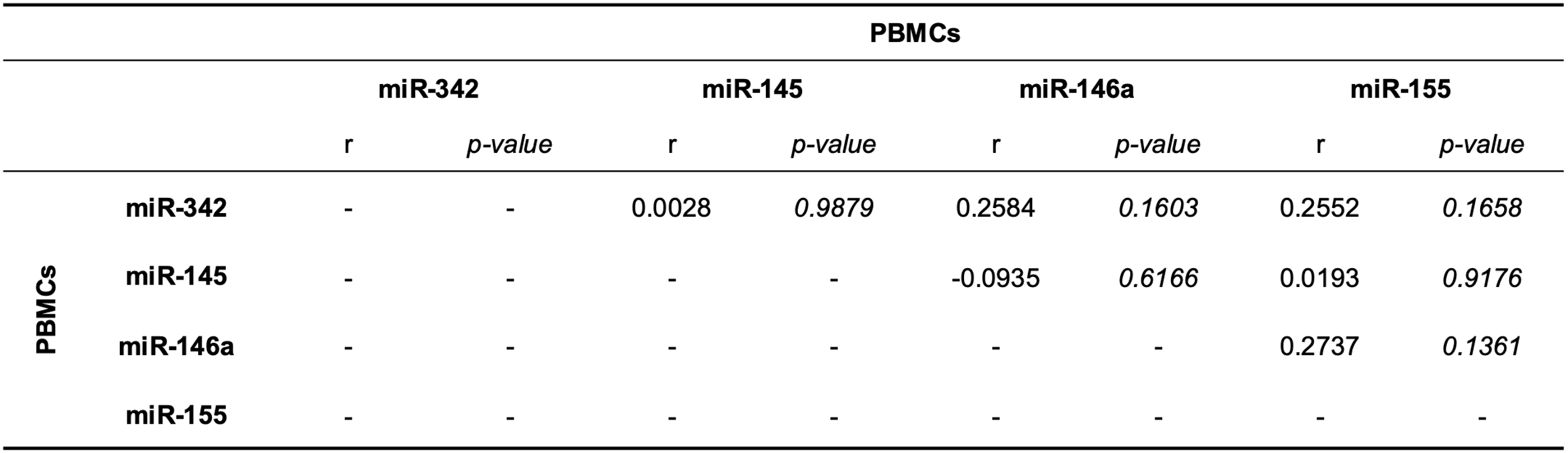


**Table S6. ROC analysis of the differently expressed miRNAs, individually or combined.** AUC – area under the curve; CI – confidence interval.


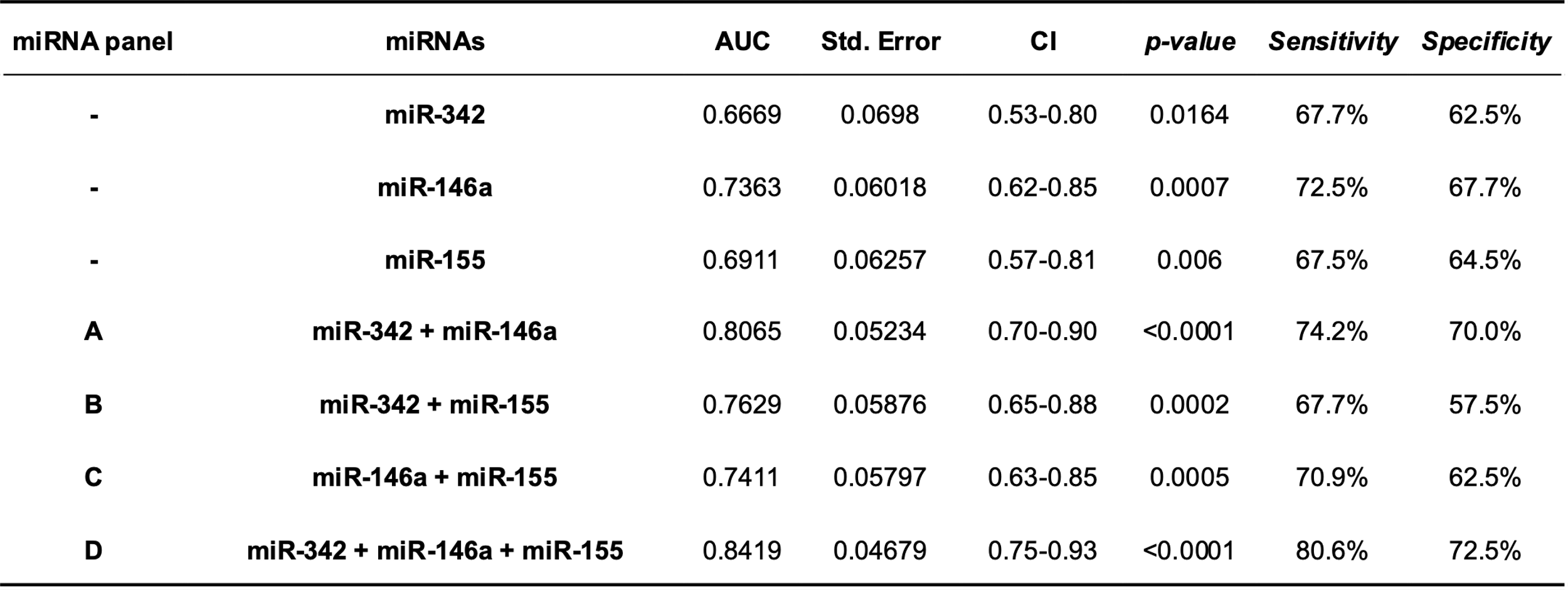

Supplement: Supplementary file 1 [file Data_Sheet_1.DOCX]
